# Supplementary material for: Identification of photoperiod-induced specific miRNAs in the adrenal glands of Sunite sheep (Ovis aries)
Source: Front Vet Sci. 2022 Jul 22;9:888207. doi: 10.3389/fvets.2022.888207 (PMC9354845; doi:10.3389/fvets.2022.888207)
Supplement: Supplementary file 1 [file Data_Sheet_1.ZIP › Supplementary file/miRDeep2 file.zip!/miRDeep2 file/Know_Structure/SP42A2/oar-mir-1185.pdf]

miRBase precursor : oar-mir-1185
Total read count : 215
oar-miR-1185-5p read count : 87
oar-miR-1185-3p read count : 128
remaining reads : 0

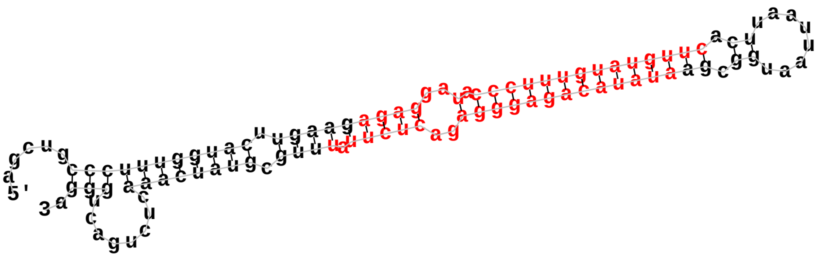

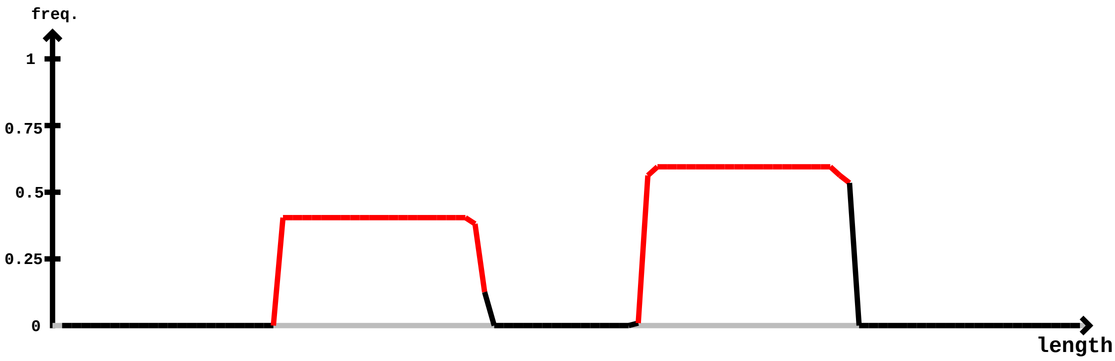

oar-miR-1185-5p

oar-miR-1185-3p

```
5'- agcugcccuuugguacuugaagagaggauacccuuuguauguucacuuaauuaauggcgaauauacagagggagacucuuauuugcguaucaaacucugacuggga   -3'    exp
    .....((((((((((.(((((((((..(.(((((((((((((((.((........)).)))))))))))))))..))))).)))).))))))))........))).    reads      mm          sample
    ......................agaggauacccuuuguaugu................................................................    5          0           seq
    ......................agaggauacccuuuguauguu...............................................................    55         0           seq
    ......................agaggauacccuuuguauguuc..............................................................    27         0           seq
    ...........................................................aauauacagagggagacucuuau........................    2          0           seq
    ............................................................auauacagagggagacucuu..........................    5          0           seq
    ............................................................auauacagagggagacucuua.........................    6          0           seq
    ............................................................auauacagagggagacucuuau........................    108        0           seq
    .............................................................uauacagagggagacucuu..........................    2          0           seq
    .............................................................uauacagagggagacucuuau........................    5          0           seq
```
